# Age- and sex-specific reference intervals and determinants of plasma vitamin B6 metabolites in healthy Chinese adults

**DOI:** 10.3389/fnut.2026.1782217

**Published:** 2026-02-17

**Authors:** Yazhao Mei, Ziyuan Wang, Li Shen, Zhenlin Zhang, Hua Yue, Hao Zhang, Jiemei Gu, Weiwei Hu, Shanshan Li, Chao Gao, Zhe Wei, Yang Xu, Jie Wang, Gao Gao, Chun Wang

**Affiliations:** 1 Shanghai Clinical Research Center of Bone Disease, Department of Osteoporosis and Bone Diseases, Shanghai Sixth People’s Hospital Affiliated to Shanghai Jiao Tong University School of Medicine, Shanghai, China; 2 Clinical Research Center, Shanghai Sixth People’s Hospital Affiliated to Shanghai Jiao Tong University School of Medicine, Shanghai, China; 3 Medical Examination Center, Shanghai Sixth People’s Hospital Affiliated to Shanghai Jiao Tong University School of Medicine, Shanghai, China

**Keywords:** 4-pyridoxic acid, multivariate linear regression, pyridoxal, pyridoxal 5′-phosphate, reference interval

## Abstract

**Objective:**

Reference intervals (RIs) for vitamin B6 biomarkers remain underexplored in Chinese adults. We aimed to establish age- and sex-specific RIs of pyridoxal 5′-phosphate (PLP), pyridoxal (PL), 4-pyridoxic acid (PA), and their ratios (PLP/PL, PLP/PA, and PAr), and to identify independent determinants of these markers.

**Methods:**

Vitamin B6 metabolites were measured by liquid chromatography–tandem mass spectrometry (LC–MS/MS). The distribution changes were illustrated by sex-stratified age percentile curves (P10/P50/P90). Robust 95% RIs (P2.5–P97.5) were obtained by sex and age (<50 vs. ≥50 years) using the Horn–Pesce method. Sex-stratified multivariable linear regression was conducted to identify the independent determinants of vitamin B6 biomarkers.

**Results:**

This study included a community-based sample of 367 healthy adults (197 males and 170 females) with a median age of 49.0 years (range 20.0–80.0 years). Females had higher PLP, PL and PLP/PA, and lower PAr than males (all *p* < 0.01). In males, percentile curves and age-group contrasts showed age-associated declines in PLP, PLP/PL and PLP/PA and an increase in PAr (all *p* < 0.05); corresponding indices in females were largely stable. The RIs of PLP (nmol/L) were 10.36–145.87 (males <50 years, *n* = 103), 13.03–103.46 (females <50 years, *n* = 87), 9.27–142.96 (males ≥50 years, *n* = 94), and 16.83–173.06 (females ≥50 years, *n* = 83), respectively. Higher ALP levels were associated with lower PLP, PLP/PL and PLP/PA, and higher PAr; albumin was positively related to PLP/PL and PLP/PA, and negatively to PAr; eGFR was positively correlated with PLP/PA, and negatively with PAr. Notably, serum phosphorus was positively associated with PLP, PLP/PL and PLP/PA, and negatively with PAr.

**Conclusion:**

We established population-specific, age- and sex-stratified RIs for vitamin B6 biomarkers in healthy Chinese adults, providing a baseline for clinical and laboratory assessment of vitamin B6 metabolism. We also found that ALP, albumin, renal function, and serum phosphorus were closely related to the vitamin B6 status in both males and females.

## 1 Introduction

Vitamin B6 status is routinely assessed using circulating pyridoxal 5′-phosphate (PLP), yet accumulating evidence suggests that single vitamers provide an incomplete picture of vitamin B6 metabolism (1–3). PLP, pyridoxal (PL), and 4-pyridoxic acid (PA) reflect distinct functional and catabolic states, and composite indices such as PLP/PL, PLP/PA, and PAr = PA/(PLP + PL) have been proposed to capture the balance between coenzyme supply and degradation (2). These ratio-based markers are considered more stable indicators of vitamin B6 functional status, being less influenced by transient dietary intake and several confounding conditions (2, 4, 5). The recent cohort studies further showed that composite indices reflecting the PLP and PA balance, including PA/PLP and PAr, predict morbidity and mortality more strongly than PLP alone (6–9), supporting the incorporation of these composite markers into the evaluation of vitamin B6 status.

However, interpretation of vitamin B6 biomarkers is complicated by their sensitivity to ethnicity, diet and supplement use, inflammation, renal dysfunction, altered alkaline phosphatase (ALP) activity, hypoalbuminemia, and other metabolic perturbations (2, 9–12). These factors can change PLP, PL, PA and related ratios independently of vitamin B6 intake, underscoring the need for robust, population-specific reference intervals (RIs) to guide clinical and epidemiologic use. Existing data are dominated by Western cohorts and focus primarily on PLP, with limited concurrent measurement of PL, PA and ratio-based indices, and scarce exploration of age- and sex-specific patterns in Asian populations (4, 10, 13, 14). Whether healthy Chinese adults exhibit distinct distributions of PLP, PL, PA and related ratios, and how these markers vary by age, sex, and key biochemical determinants, remain largely unknown. Importantly, individuals with reduced ALP activity, such as those with hypophosphatasia (HPP), caused by loss of function mutations of *ALPL* gene, are characterized by significantly elevated circulating PLP levels (15–17). Establishing RIs of vitamin B6, especially for PLP, may therefore facilitate earlier identification of HPP.

Therefore, we conducted a community-based study of rigorously screened healthy Chinese adults with two objectives: (1) to establish age- and sex-specific RIs for PLP, PL, PA and their ratios (PLP/PL, PLP/PA, PAr) using the methods recommended by the CLSI EP28-A3c guidelines; and (2) to identify independent biochemical determinants of vitamin B6 biomarkers.

## 2 Materials and methods

### 2.1 Study design and participants

This study was approved by the Ethics Committee of Shanghai Sixth People’s Hospital affiliated to Shanghai Jiao Tong University School of Medicine (Approved no. 2024-KY-135(K)). Written informed consent was obtained from all participants prior to enrollment.

A community-based sample of adults (aged ≥18 years) in Shanghai was recruited from July to October 2024 to establish age- and sex-specific RIs for plasma vitamin B6 metabolites. Detailed demographic, anthropometric, and clinical data were collected, including age, sex, height, weight, body mass index (BMI), and blood pressure. Each participant underwent a standardized health examination and completed a validated questionnaire addressing lifestyle behaviors, smoking and drinking habits, and current medication use. Participants were excluded if they were pregnant or lactating, had BMI ≥ 30 kg/m^2^ or <18.5 kg/m^2^, were current smokers, or had alcohol use disorder, anemia, hypertension, diabetes mellitus, dyslipidemia, hypoalbuminemia (<35 g/L), chronic kidney (eGFR <60 mL/min/1.73 m^2^) or liver disease, abnormal ALP levels (<40 U/L or >125 U/L), systemic inflammation (CRP ≥ 5 mg/L), active malignancy, thyroid dysfunction, or recent vitamin B6 supplementation within 1 week.

### 2.2 Biochemical measurements

Fasting venous blood samples were collected between 8:00 and 10:00 a.m. following an overnight fast and protected from light until analysis. All measurements were performed in a single batch using the same reagent lot and following standardized laboratory quality control protocols. Complete blood count (CBC) and C-reactive protein (CRP) were analyzed using an AU5811 hematology analyzer (Beckman Coulter, Brea, CA, USA). Serum levels of calcium, phosphorus, alanine aminotransferase (ALT), ALP, creatinine, uric acid, total cholesterol (TC), triglycerides (TG), and glycated hemoglobin (HbA1c) were measured with a Hitachi 7,600 automatic biochemical analyzer (Hitachi Ltd., Tokyo, Japan). The estimated GFR (eGFR) was calculated using the creatinine-based Chronic Kidney Disease Epidemiology Collaboration (CKD-EPI) equation (18). Reference ranges were obtained from the Department of Laboratory Medicine, Shanghai Sixth People’s Hospital.

Plasma levels of PLP, PL and PA were quantified using liquid chromatography–tandem mass spectrometry (LC–MS/MS) with the Diasis Water-Soluble Vitamin Sample Extraction Kit (Zhejiang Diasis Diagnostic Technologies Co., Ltd., China), following the manufacturer’s protocol. For PLP, intra-assay coefficients of variation (CVs) were 6.22% (LQC) and 4.73% (HQC), and inter-assay CVs were 7.85% (LQC) and 5.23% (MQC). For PL, intra-assay CVs were 1.07% (LQC) and 1.34% (HQC), and inter-assay CVs were 2.62% (LQC) and 2.52% (MQC). For PA, intra-assay CVs were 3.77% (LQC) and 4.11% (HQC), and inter-assay CVs were6.39% (LQC) and 3.13% (MQC).

### 2.3 Statistical analysis

Continuous variables were tested for normality using the Shapiro–Wilk test. Variables with an approximately normal distribution were presented as mean ± standard deviation and were compared using independent-samples *t* tests or one-way ANOVA, with Levene’s test applied to assess homogeneity of variance. Non-normally distributed variables were reported as median (Q1, Q3) and were compared using the Mann–Whitney *U* test or Kruskal–Wallis’s test, as appropriate.

Prior to RI establishment, we evaluated potential outliers within each subgroup (sex and age group) using Tukey’s interquartile-range (IQR) fences (1.5 × IQR for mild and 3 × IQR for extreme values). Then, based on the analysis of quality control records, raw data, consistency with exclusion criteria, and possible supplement use or recent medical history, each value marked as an outlier was individually reviewed. Due to the absence of evidence of measurement errors, sample handling issue, or violations of exclusion criteria, all values were retained. RIs were established using CLSI EP28-A3c-recommended robust methods to mitigate the impact of extreme values.

Sex-specific age–percentile curves (P10/P50/P90) were generated to visualize distributional shifts across adulthood. RIs for vitamin B6 metabolites were established after stratification by age (<50 vs. ≥50 years) and sex. The cutoff age of 50 was mainly based on the following four points: Firstly, it was close to the median age of our cohort (49 years old). Secondly, a previous study establishing age- and sex-specific RIs of PLP levels also used 50 years as the cutoff. Thirdly, we found that the age of 50 was the point where significant changes occurred in the sex-specific percentile curves. Fourthly, the age of 50 is widely recognized as the midlife boundary, which is associated with physiological changes such as menopausal hormone changes (19). The robust method described by Horn and Pesce (20) was applied to estimate 95% RIs (P2.5–P97.5) due to the sample sizes of subgroups below 120, in accordance with the CLSI EP28-A3c guideline. The stability of the estimated intervals was further verified using nonparametric bootstrap resampling.

To explore factors associated with vitamin B6 metabolites, multivariate linear regression by sex was performed. Outcome variables (e.g., PLP, PLP/PL) were naturally log-transformed to meet model assumptions. Covariates included age, BMI, ALP, ALT, eGFR, CRP, calcium, phosphorus, and albumin.

All statistical analyses were conducted using R version 4.2.2 (R Foundation for Statistical Computing, Vienna, Austria), with a two-tailed *p* value < 0.05 considered statistically significant.

## 3 Results

### 3.1 Characteristics of the study population

**Figure 1:**
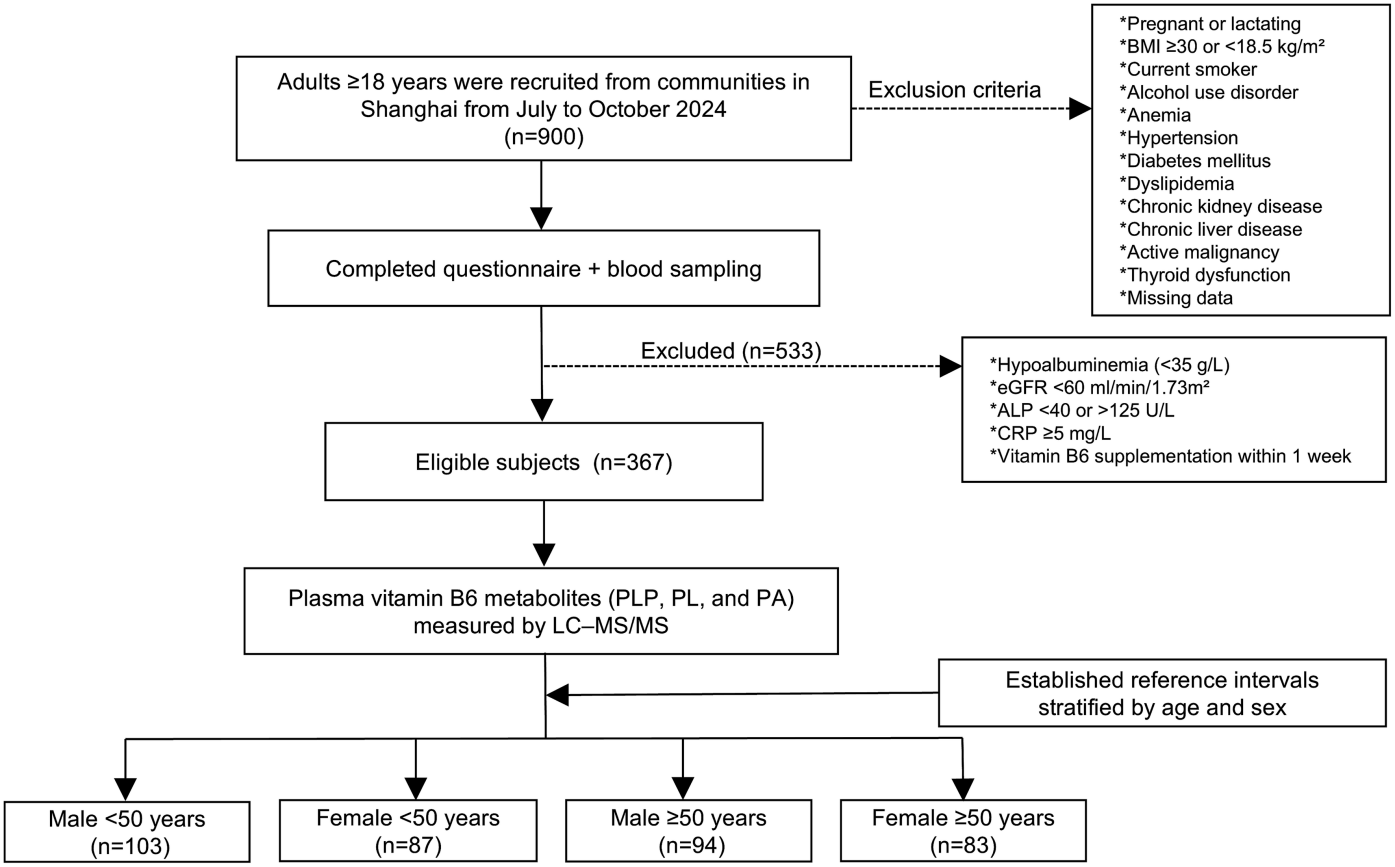
Study flowchart. BMI, body mass index; ALP, alkaline phosphatase; CRP, C-reactive protein; PLP, pyridoxal 5′-phosphate; PL, pyridoxal; PA, 4-pyridoxic acid; LC–MS/MS, liquid chromatography–tandem mass spectrometry.

This study included 367 healthy individuals (197 males and 170 females) with a median age of 49.0 years (range 20.0–80.0 years) (Figure 1). Baseline characteristics by sex were shown in Table 1. The age between males and females did not differ (49.0 (Q1, Q3: 32.0, 60.0) years vs. 48.0 (Q1, Q3: 35.0, 61.0) years; *p* = 0.617). Significant sex-related differences were observed in height, weight, BMI, SBP, DBP, cholesterol, albumin, ALP, HGB, ALT, eGFR, uric acid, and phosphorus levels (all *p* < 0.05). Females exhibited significantly higher levels of PLP, PL and PLP/PA, and lower PAr compared to males (all *p* < 0.01), whereas PA and PLP/PL levels were comparable between sex.

**Table 1:** Baseline characteristics by sex in the healthy population.

Variable	Total (*n* = 367)	Male (*n* = 197)	Female (*n* = 170)	*p* value
Age (years)	49.0 (33.0, 60.0)	49.0 (32.0, 60.0)	48.0 (35.0, 61.0)	0.617
Height (cm)	167.2 (162.0, 174.8)	174.0 (170.0, 178.3)	161.8 (157.5, 165.2)	**<0.001**
Weight (kg)	63.3 (55.6, 72.0)	70.3 (64.2, 76.2)	56.0 (51.2, 60.8)	**<0.001**
BMI (kg/m^2^)	22.5 (20.6, 24.5)	23.5 (21.7, 25.4)	21.6 (19.7, 23.5)	**<0.001**
SBP (mmHg)	123.0 (111.0, 136.0)	126.0 (116.0, 136.0)	117.0 (106.0, 134.0)	**0.001**
DBP (mmHg)	75.0 (69.0, 83.0)	78.0 (72.0, 85.0)	72.0 (65.0, 78.0)	**<0.001**
Cholesterol (mmol/L)	4.9 (4.4, 5.5)	4.8 (4.2, 5.3)	5.0 (4.6, 5.7)	**<0.001**
Triglyceride (mmol/L)	1.1 (0.8, 1.6)	1.1 (0.8, 1.6)	1.0 (0.8, 1.6)	**0.034**
CRP (mg/L)	0.5 (0.2, 0.9)	0.5 (0.2, 1.0)	0.5 (0.2, 0.9)	0.745
HGB (g/L)	144.5 ± 14.8	154.4 ± 10.6	132.8 ± 9.5	**<0.001**
Albumin (g/L)	45.9 ± 2.4	46.3 ± 2.5	45.3 ± 2.3	**<0.001**
ALT (U/L)	18.0 (13.0, 25.0)	20.0 (15.0, 28.0)	15.5 (12.0, 21.2)	**<0.001**
HbA1c (%)	5.50 (5.20, 5.80)	5.50 (5.20, 5.80)	5.40 (5.20, 5.70)	0.542
eGFR (mL/min/1.73^2^)	94.7 (85.0, 104.4)	92.9 (85.0, 101.0)	97.6 (85.8, 107.0)	**<0.001**
Uric acid (μmol/L)	341.0 (285.5, 396.5)	387.0 (336.5, 426.0)	291.5 (252.8, 338.2)	**<0.001**
Calcium (mmol/L)	2.37 (2.30, 2.43)	2.4 (2.3, 2.4)	2.37 (2.32, 2.43)	0.489
Phosphorus (mmol/L)	1.08 ± 0.14	1.03 ± 0.14	1.14 ± 0.11	**<0.001**
ALP (U/L)	69.0 (59.0, 82.0)	71.0 (63.0, 82.0)	66.0 (55.8, 82.0)	**0.006**
PLP (nmol/L)	35.48 (26.07, 49.24)	32.47 (24.86, 46.11)	39.24 (28.65, 50.36)	**0.004**
PL (nmol/L)	14.91 (11.63, 18.05)	14.12 (11.15, 16.73)	15.67 (12.49, 18.96)	**0.003**
PA (nmol/L)	15.79 (12.81, 20.26)	16.18 (13.16, 19.80)	15.29 (12.50, 20.81)	0.959
PLP/PL	2.42 (2.01, 2.87)	2.37 (1.97, 2.89)	2.51 (2.10, 2.84)	0.216
PLP/PA	2.18 (1.67, 3.05)	1.99 (1.61, 2.87)	2.37 (1.75, 3.23)	**0.003**
PAr	0.32 (0.24, 0.41)	0.34 (0.25, 0.43)	0.30 (0.23, 0.39)	**0.003**

Normally distributed variables were compared using independent *t*-tests and reported as mean ± standard deviation, while non-normally distributed variables were analyzed with Mann–Whitney *U* tests and summarized as median (Q1, Q3). Categorical variables were presented as frequency (percentage) and analyzed using Pearson’s chi-square. Statistical significance was defined as two-tailed *p* < 0.05, with significant results bold. PAr = PA/(PLP + PL). Normal reference ranges for indices: Cholesterol: 2.80–5.90 mmol/L; Triglyceride: 0.45–1.81 mmol/L; CRP: 0–5 mg/L; HGB: 115–150 g/L; Albumin:35.0–55.0 g/L; ALT: 0–65 U/L; HbA1c: 4.3–6.5%; eGFR ≥ 60 mL/min/1.73^2^; Uric acid: 210–430 μmol/L; serum calcium: 2.08–2.60 mmol/L; serum phosphorus: 0.80–1.60 mmol/L; ALP: 45–125 U/L (Male), 35–100 U/L (Female). BMI, body mass index; SBP, systolic blood pressure; DBP, diastolic blood pressure; CRP, C-reactive protein; HGB, hemoglobin; ALT, alanine aminotransferase; HbA1c, glycated hemoglobin; ALP, alkaline phosphatase; PLP, pyridoxal 5′-phosphate; PL, pyridoxal; PA, 4-pyridoxic acid.

### 3.2 Age- and sex-related variations and RIs of vitamin B6 metabolites

The sex-stratified age-percentile curves (P10/P50/P90) revealed distinct age-related patterns of plasma PLP, PL and PA levels, as well as their ratios (PLP/PL, PLP/PA, PAr) (Supplementary Figure 1). After grouping by age (<50 years and ≥50 years), inter group comparisons were conducted for males and females, respectively (Table 2). In males, PLP in ≥50-year-old group was significantly lower than that in the <50-year-old group (*p* = 0.039). PA showed a slight and insignificant age-related trend, while PL remained relatively stable across different age groups. Consistent with the curve, the decrease in PLP/PL and PLP/PA, as well as the increase in PAr, were more significant in those aged ≥50 years (all *p* < 0.01). In females, PL and PA increased slightly with age and the levels in ≥50-year-old group were significantly higher than those in <50-year-old group (both *p* < 0.01). However, there were no significant differences in PLP, PLP/PL, PLP/PA, and PAr between the two age groups (all *p* > 0.05). These distribution changes suggest that RIs need to be stratified by age and sex.

**Table 2:** Sex-stratified comparisons of vitamin B6 biomarkers between participants aged <50 and ≥50 years.

Variable	Males			Females		
	<50 years	≥50 years	*p* value	<50 years	≥50 years	*p* value
PLP (nmol/L)	38.44 (26.22, 50.54)	31.68 (23.91, 40.54)	**0.039**	39.65 (27.43, 56.85)	41.38 (31.08, 50.17)	0.185
PL (nmol/L)	14.66 (11.25, 17.65)	14.00 (11.01, 16.27)	0.457	14.78 (11.61, 17.95)	16.99 (14.24, 19.92)	**0.002**
PA (nmol/L)	15.67 (12.94, 18.07)	17.25 (13.76, 21.35)	0.080	15.23 (11.68, 18.84)	18.45 (14.14, 22.87)	**0.001**
PLP/PL	2.59 ± 0.76	2.29 ± 0.56	**0.002**	2.57 ± 0.57	2.42 ± 0.56	0.097
PLP/PA	2.33 (1.69, 3.11)	1.91 (1.40, 2.47)	**0.001**	2.45 (1.79, 3.30)	2.30 (1.74, 3.11)	0.271
PAr	0.31 (0.24, 0.40)	0.37 (0.29, 0.46)	**0.002**	0.30 (0.22, 0.37)	0.31 (0.24, 0.39)	0.373

Statistical significance was defined as two-tailed *p* < 0.05, with significant results bold. PAr = PA/(PLP + PL). PLP, pyridoxal 5′-phosphate; PL, pyridoxal; PA, 4-pyridoxic acid.

The 95% RIs (P2.5–P97.5) for six vitamin B6–related biomarkers (PLP, PL, PA, PLP/PL, PLP/PA, and PAr), stratified by sex and age group (<50 years and ≥50 years), were summarized in Table 3. Sample sizes were 103 for males <50 years, 87 for females <50 years, 94 for males ≥50 years, and 83 for females ≥50 years, respectively.

**Table 3:** Reference intervals of vitamin B6 metabolites stratified by age and sex.

Variable	Age group	Sex	*n*	Median (Q1, Q3)	P2.5 (95%CI)	P97.5 (95%CI)
PLP (nmol/L)	<50 years	Male	103	38.44 (26.22, 50.54)	10.36 (7.49–16.91)	145.87 (84.12–150.60)
		Female	87	39.65 (27.43, 56.85)	13.03 (7.08–17.03)	103.46 (71.05–115.04)
	≥50 years	Male	94	31.68 (23.91, 40.54)	9.27 (8.21–14.16)	142.96 (79.11–166.95)
		Female	83	41.38 (31.08, 50.17)	16.83 (15.58–20.11)	173.06 (122.80–189.85)
PL (nmol/L)	<50 years	Male	103	14.66 (11.25, 17.65)	7.18 (6.10–8.61)	49.95 (26.74–66.34)
		Female	87	14.78 (11.61, 17.95)	7.48 (7.12–9.33)	37.87 (24.65–52.11)
	≥50 years	Male	94	14.00 (11.01, 16.27)	6.58 (5.08–8.14)	54.62 (30.87–65.87)
		Female	83	16.99 (14.24, 19.92)	7.78 (6.34–9.99)	71.43 (37.75–72.74)
PA (nmol/L)	<50 years	Male	103	15.67 (12.94, 18.07)	8.19 (6.39–10.21)	47.66 (32.32–67.21)
		Female	87	15.23 (11.68, 18.84)	8.03 (5.84–8.68)	35.60 (27.52–44.61)
	≥50 years	Male	94	17.25 (13.76, 21.35)	9.23 (7.53–10.26)	46.35 (31.34–66.11)
		Female	83	18.45 (14.14, 22.87)	9.34 (7.32–11.25)	52.90 (34.72–54.43)
PLP/PL	<50 years	Male	103	2.57 (1.99, 3.17)	1.16 (0.93-1.48)	4.23 (3.88-4.66)
		Female	87	2.55 (2.18, 2.88)	1.47 (0.97-1.77)	4.06 (3.42-4.18)
	≥50 years	Male	94	2.28 (1.90, 2.68)	1.27 (1.17-1.39)	3.34 (3.18-4.09)
		Female	83	2.41 (1.96, 2.78)	1.39 (1.35-1.59)	3.56 (3.37-3.60)
PLP/PA	<50 years	Male	103	2.42 (1.68, 3.12)	0.76 (0.59-1.14)	5.15 (4.35-5.45)
		Female	87	2.60 (1.74, 3.33)	1.27 (0.88-1.41)	5.73 (4.51-7.62)
	≥50 years	Male	94	1.96 (1.38, 2.49)	0.57 (0.43-1.00)	3.99 (3.46-6.12)
		Female	83	2.41 (1.72, 3.13)	1.08 (0.95-1.37)	4.76 (3.79-5.73)
PAr	<50 years	Male	103	0.32 (0.24, 0.40)	0.15 (0.15-0.18)	0.69 (0.58-0.86)
		Female	87	0.30 (0.22, 0.37)	0.14 (0.10-0.17)	0.54 (0.48-0.56)
	≥50 years	Male	94	0.38 (0.28, 0.47)	0.19 (0.13-0.21)	1.03 (0.66-1.37)
		Female	83	0.32 (0.24, 0.39)	0.16 (0.13-0.19)	0.57 (0.47-0.61)

The robust method based on Horn’s algorithm was used to calculate 95% reference intervals (P2.5–P97.5), and their stability was validated via nonparametric bootstrap resampling. PLP, pyridoxal 5′-phosphate; PL, pyridoxal; PA, 4-pyridoxic acid.

### 3.3 Factors associated with vitamin B6 metabolites

**Figure 2:**
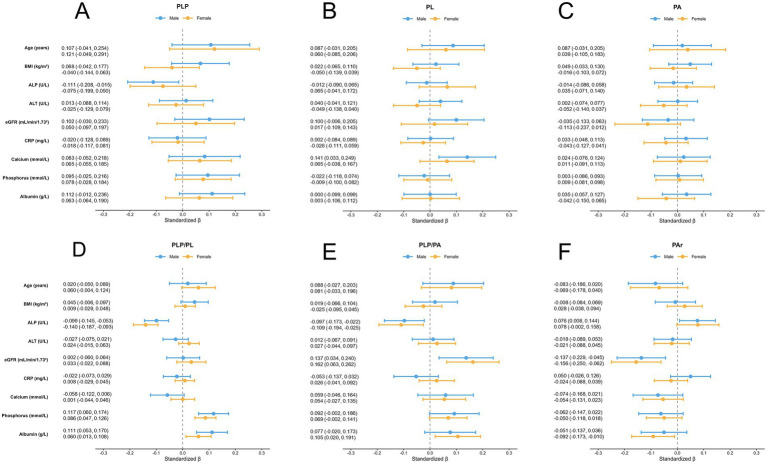
Sex-stratified multivariable linear regression forest plot of vitamin B6 biomarkers. The regression coefficients (standardized *β* values) and 95% confidence intervals (CIs) are presented for each variable. **(A)** PLP, **(B)** PL, **(C)** PA, **(D)** PLP/PL, **(E)** PLP/PA, and **(F)** PAr. BMI, body mass index, ALP, alkaline phosphatase, ALT, alanine aminotransferase; CRP, C-reactive protein; PLP, pyridoxal 5′-phosphate; PL, pyridoxal; PA, 4-pyridoxic acid.

Sex-stratified multivariate linear regression was conducted to identify independent determinants of vitamin B6 metabolites (Figure 2; Supplementary Tables 1 and 2), and effect directions were broadly similar in males and females. The serum ALP level was negatively correlated with plasma PLP levels and PLP based ratios (PLP/PL, PLP/PA), whereas positively correlated with PAr. The serum albumin level was positively correlated with PLP/PL and PLP/PA, and negatively correlated with PAr. Higher eGFR was associated with lower PAr and higher PLP/PA, while serum phosphorus levels were positively correlated with PLP and their ratios (PLP/PL, PLP/PA), and negatively correlated with PAr. However, age, BMI, ALT, CRP, and calcium showed non-significant associations with vitamin B6 metabolites after multivariable adjustment.

## 4 Discussion

In this community-based sample of healthy Chinese adults, we established age- and sex- specific RIs for plasma vitamin B6 metabolites (PLP, PL, PA) and their ratios (PLP/PL, PLP/PA, PAr) using guideline-recommended robust methods, characterized age trends with sex-stratified percentile curves, and examined independent correlates in sex-stratified multivariable models. The percentile curves demonstrated clear age-associated distributional changes, supporting the use of sex- and age-specific RIs (<50 vs. ≥ 50 years) for clinical interpretation.

In this study, we found that vitamin B6 metabolism differed by age and sex, and the age-related decline in PLP was limited to males. This finding is consistent with the NHANES study on PLP levels in American adults (10). However, compared with American adults, the plasma PLP levels in our cohort was lower in males and higher in females. Additional analysis from NHANES also clarified the existence of race-related differences in PLP levels (10). These results suggest that race is an important factor affecting vitamin B6 homeostasis, and the RIs derived from Western populations cannot be directly applied to Chinese adults. Therefore, it is necessary to establish Chinese-specific RIs to ensure accurate assessment and clinical interpretation of vitamin B6 status. In our cohort, the median PLP concentration of all subgroups was above 30 nmol/L, which is the conventional adequacy threshold used to define the status of vitamin B6 sufficiency (1), indicating that our population is unlikely to exhibit significant PLP deficiency.

Our multivariate linear regression analyses found a strong correlation between ALP, eGFR, albumin, and phosphorus and vitamin B6 metabolites in both males and females. Specifically, serum ALP levels were strongly negatively correlated with plasma PLP levels and PLP based ratios, and positively correlated with PAr, which is consistent with the role of ALP in dephosphorylation from PLP to PL (2). Individuals with HPP are characterized by significantly elevated circulating PLP levels (15–17). In contrast, children diagnosed with familial hypophosphatemic rickets exhibit elevated levels of ALP and significantly reduced plasma PLP (21). Albumin was positively associated with PLP/PL and PLP/PA and inversely with PAr, supporting its function as a major PLP-binding protein and suggesting that reduced albumin may promote a more catabolic vitamin B6 profile (3). In previous studies, higher creatinine or lower eGFR were associated with increased PA and PAr, as well as decreased PLP (9, 22), which is consistent with the renal clearance of PA and our findings that renal function indicators shape PLP-PA balance.

It is worth noting that our study found a novel and independent association between serum level of phosphorus and vitamin B6 metabolism. The serum level of phosphorus was positively correlated with PLP, PLP/PL and PLP/PA, and negatively correlated with PAr. So far, the clinical connection between these two parameters is still unclear. We believe that this phenomenon can be explained by the regulatory mechanism of ALP activity. Inorganic phosphate is a recognized competitive inhibitor that affects ALP activity (23, 24). Therefore, higher physiological levels of serum phosphorus may lead to downregulation of ALP activity, thereby inhibiting ALP mediated degradation of PLP, and hindering cellular uptake of PLP, resulting in the accumulation of PLP in the plasma. Further mechanistic and longitudinal studies are needed to confirm whether the observed association represents a genuine physiological interaction between phosphorus homeostasis and vitamin B6 metabolism.

Based on these determinants, our sex-stratified age analyses revealed distinct patterns of vitamin B6 metabolism. In males, age-percentile curves and group comparisons (<50 vs. ≥ 50 years) showed lower PLP and PLP-based ratios and higher PAr in older compared with younger individuals, indicating a higher vitamin B6 turnover rate (6, 7). Older males also exhibited lower albumin, eGFR, and phosphorus (Supplementary Table 3), aligning with our regression results: reduced protein binding, diminished renal clearance capacity, and lower serum phosphorus together favor a more catabolic PLP–PA balance. In females, by contrast, PLP and related ratios remained relatively stable across all age groups, suggesting that sex-specific differences in hormonal or metabolic factors may attenuate age-related shifts observed in males. Collectively, these findings provide a mechanistic rationale for age- and sex-specific RIs and remind people not to generalize cutoff values that may misclassify elderly men or younger women.

This study has several limitations. Firstly, although strict exclusion criteria ensured internal validity, the overall and subgroup sample sizes were modest. Therefore, we applied Horn’s robust method, which is recommended by CLSI for small to medium samples and minimizes the influence of outliers. Nevertheless, moderate sizes can lead to wider confidence intervals for the estimated reference limits, particularly for upper limits, introducing statistical uncertainty. Larger scale and multicenter studies are warranted to validate and refine these RIs across diverse Chinese populations in the future. Secondly, we did not evaluate dietary vitamin B6 intake, the status of vitamin D, parathyroid hormone, or bone turnover markers, which may affect both ALP activity and vitamin B6 biomarkers (25, 26). Thirdly, the participants in this study were from Shanghai. Regional differences in dietary patterns, lifestyles, and socioeconomic status across China may affect the status and metabolism of vitamin B6. Therefore, external validation is still needed in multicenter cohorts from different regions. Lastly, previous prospective studies have linked high-turnover B6 profiles to adverse outcomes; the elderly males in our cohort exhibited such a pattern, suggesting a potentially less favorable phenotype. However, given the cross-sectional design and sex–age differences in eGFR, albumin, and mineral indices, this observation needs to be prospectively validated in the Chinese population.

## 5 Conclusion

In summary, we have established age- and sex-specific RIs for plasma PLP, PL, PA, and related ratios (PLP/PL, PLP/PA, and PAr) in healthy Chinese adults through rigorous screening, which can provide a reference baseline for clinical and laboratory evaluation of vitamin B6 metabolism. Meanwhile, a higher turnover rate of vitamin B6 was observed in elderly males. Serum ALP, albumin, renal function, and serum phosphorus are the four major factors significantly correlated with vitamin B6 status. Given the cross-sectional design of our study, the clinical prognostic significance of the high turnover vitamin B6 phenotype requires further validation in prospective cohorts of the Chinese population.

## Data Availability

The original contributions presented in the study are included in the article/Supplementary material, further inquiries can be directed to the corresponding author/s.
