# Correction: Age- and sex-specific reference intervals and determinants of plasma vitamin B6 metabolites in healthy Chinese adults

**DOI:** 10.3389/fnut.2026.1834865

**Published:** 2026-04-21

**Authors:** Yazhao Mei, Ziyuan Wang, Li Shen, Zhenlin Zhang, Hua Yue, Hao Zhang, Jiemei Gu, Weiwei Hu, Shanshan Li, Chao Gao, Zhe Wei, Yang Xu, Jie Wang, Gao Gao, Chun Wang

**Affiliations:** 1 Shanghai Clinical Research Center of Bone Disease, Department of Osteoporosis and Bone Diseases, Shanghai Sixth People's Hospital Affiliated to Shanghai Jiao Tong University School of Medicine, Shanghai, China; 2 Clinical Research Center, Shanghai Sixth People's Hospital Affiliated to Shanghai Jiao Tong University School of Medicine, Shanghai, China; 3 Medical Examination Center, Shanghai Sixth People's Hospital Affiliated to Shanghai Jiao Tong University School of Medicine, Shanghai, China

**Keywords:** 4-pyridoxic acid, multivariate linear regression, pyridoxal, pyridoxal 5′-phosphate, reference interval

There were mistakes in Tables 1–3 as published. We identified some errors in the calculation of derived ratio indices (PLP/PL, PLP/PA, PAr) reported in the manuscript. The corrected Tables 1–3 appears below.

**Table 1:** Baseline characteristics by sex in the healthy population.

Variable	Total (*n* = 367)	Male (*n* = 197)	Female (*n* = 170)	*P* value
Age (years)	49.0 (33.0, 60.0)	49.0 (32.0, 60.0)	48.0 (35.0, 61.0)	0.617
Height (cm)	167.2 (162.0, 174.8)	174.0 (170.0, 178.3)	161.8 (157.5, 165.2)	**< 0.001**
Weight (kg)	63.3 (55.6, 72.0)	70.3 (64.2, 76.2)	56.0 (51.2, 60.8)	**< 0.001**
BMI (kg/m^2^)	22.5 (20.6, 24.5)	23.5 (21.7, 25.4)	21.6 (19.7, 23.5)	**< 0.001**
SBP (mmHg)	123.0 (111.0, 136.0)	126.0 (116.0, 136.0)	117.0 (106.0, 134.0)	**0.001**
DBP (mmHg)	75.0 (69.0, 83.0)	78.0 (72.0, 85.0)	72.0 (65.0, 78.0)	**< 0.001**
Cholesterol (mmol/L)	4.9 (4.4, 5.5)	4.8 (4.2, 5.3)	5.0 (4.6, 5.7)	**< 0.001**
Triglyceride (mmol/L)	1.1 (0.8, 1.6)	1.1 (0.8, 1.6)	1.0 (0.8, 1.6)	**0.034**
CRP (mg/L)	0.5 (0.2, 0.9)	0.5 (0.2, 1.0)	0.5 (0.2, 0.9)	0.745
HGB (g/L)	144.5 ± 14.8	154.4 ± 10.6	132.8 ± 9.5	**< 0.001**
Albumin (g/L)	45.9 ± 2.4	46.3 ± 2.5	45.3 ± 2.3	**< 0.001**
ALT (U/L)	18.0 (13.0, 25.0)	20.0 (15.0, 28.0)	15.5 (12.0, 21.2)	**< 0.001**
HbA1c (%)	5.50 (5.20, 5.80)	5.50 (5.20, 5.80)	5.40 (5.20, 5.70)	0.542
eGFR (mL/min/1.73^2^)	94.7 (85.0, 104.4)	92.9 (85.0, 101.0)	97.6 (85.8, 107.0)	**< 0.001**
Uric acid (μmol/L)	341.0 (285.5, 396.5)	387.0 (336.5, 426.0)	291.5 (252.8, 338.2)	**< 0.001**
Calcium (mmol/L)	2.37 (2.30, 2.43)	2.4 (2.3, 2.4)	2.37 (2.32, 2.43)	0.489
Phosphorus (mmol/L)	1.08 ± 0.14	1.03 ± 0.14	1.14 ± 0.11	**< 0.001**
ALP (U/L)	69.0 (59.0, 82.0)	71.0 (63.0, 82.0)	66.0 (55.8, 82.0)	**0.006**
PLP (nmol/L)	35.48 (26.07, 49.24)	32.47 (24.86, 46.11)	39.24 (28.65, 50.36)	**0.004**
PL (nmol/L)	14.91 (11.63, 18.05)	14.12 (11.15, 16.73)	15.67 (12.49, 18.96)	**0.003**
PA (nmol/L)	15.79 (12.81, 20.26)	16.18 (13.16, 19.80)	15.29 (12.50, 20.81)	0.959
PLP/PL	2.42 (2.01, 2.87)	2.37 (1.97, 2.89)	2.51 (2.10, 2.84)	0.216
PLP/PA	2.18 (1.67, 3.05)	1.99 (1.61, 2.87)	2.37 (1.75, 3.23)	**0.003**
PAr	0.32 (0.24, 0.41)	0.34 (0.25, 0.43)	0.30 (0.23, 0.39)	**0.003**

Normally distributed variables were compared using independent t-tests and reported as mean ± standard deviation, while non-normally distributed variables were analyzed with Mann–Whitney U-tests and summarized as median (Q1, Q3). Categorical variables were presented as frequency (percentage) and analyzed using Pearson's chi-square. Statistical significance was defined as two-tailed P < 0.05, with significant results bold. PAr = PA/(PLP + PL). Normal reference ranges for indices: Cholesterol: 2.80–5.90 mmol/L; Triglyceride: 0.45–1.81 mmol/L; CRP: 0–5 mg/L; HGB: 115–150 g/L; Albumin: 35.0–55.0 g/L; ALT: 0–65 U/L; HbA1c: 4.3%−6.5%; eGFR ≥ 60 mL/min/1.73^2^; Uric acid: 210–430 μmol/L; serum calcium: 2.08–2.60 mmol/L; serum phosphorus: 0.80–1.60 mmol/L; ALP: 45–125 U/L (male), 35–100 U/L (female).

BMI, body mass index; SBP, systolic blood pressure; DBP, diastolic blood pressure; CRP, C-reactive protein; HGB, hemoglobin; ALT, alanine aminotransferase; HbA1c, glycated hemoglobin; ALP, alkaline phosphatase; PLP, pyridoxal 5′-phosphate; PL, pyridoxal; PA, 4-pyridoxic acid.

**Table 2:** Sex-stratified comparisons of vitamin B6 biomarkers between participants aged < 50 and ≥50 years.

Variable	Males			Females		
	< 50 years	≥50 years	*P* value	< 50 years	≥50 years	*P* value
PLP (nmol/L)	38.44 (26.22, 50.54)	31.68 (23.91, 40.54)	**0.039**	39.65 (27.43, 56.85)	41.38 (31.08, 50.17)	0.185
PL (nmol/L)	14.66 (11.25, 17.65)	14.00 (11.01, 16.27)	0.457	14.78 (11.61, 17.95)	16.99 (14.24, 19.92)	**0.002**
PA (nmol/L)	15.67 (12.94, 18.07)	17.25 (13.76, 21.35)	0.080	15.23 (11.68, 18.84)	18.45 (14.14, 22.87)	**0.001**
PLP/PL	2.59 ± 0.76	2.29 ± 0.56	**0.002**	2.57 ± 0.57	2.42 ± 0.56	0.097
PLP/PA	2.33 (1.69, 3.11)	1.91 (1.40, 2.47)	**0.001**	2.45 (1.79, 3.30)	2.30 (1.74, 3.11)	0.271
PAr	0.31 (0.24, 0.40)	0.37 (0.29, 0.46)	**0.002**	0.30 (0.22, 0.37)	0.31 (0.24, 0.39)	0.373

Statistical significance was defined as two-tailed P < 0.05, with significant results bold. PAr = PA/(PLP + PL).

PLP, pyridoxal 5′-phosphate; PL, pyridoxal; PA, 4-pyridoxic acid.

**Table 3:** Reference intervals of vitamin B6 metabolites stratified by age and sex.

Variable	Age group	Sex	*n*	Median (Q1, Q3)	P2.5 (95% CI)	P97.5 (95% CI)
PLP (nmol/L)	< 50 years	Male	103	38.44 (26.22, 50.54)	10.36 (7.49–16.91)	145.87 (84.12–150.60)
		Female	87	39.65 (27.43, 56.85)	13.03 (7.08–17.03)	103.46 (71.05–115.04)
	≥50 years	Male	94	31.68 (23.91, 40.54)	9.27 (8.21–14.16)	142.96 (79.11–166.95)
		Female	83	41.38 (31.08, 50.17)	16.83 (15.58–20.11)	173.06 (122.80–189.85)
PL (nmol/L)	< 50 years	Male	103	14.66 (11.25, 17.65)	7.18 (6.10–8.61)	49.95 (26.74–66.34)
		Female	87	14.78 (11.61, 17.95)	7.48 (7.12–9.33)	37.87 (24.65–52.11)
	≥50 years	Male	94	14.00 (11.01, 16.27)	6.58 (5.08–8.14)	54.62 (30.87–65.87)
		Female	83	16.99 (14.24, 19.92)	7.78 (6.34–9.99)	71.43 (37.75–72.74)
PA (nmol/L)	< 50 years	Male	103	15.67 (12.94, 18.07)	8.19 (6.39–10.21)	47.66 (32.32–67.21)
		Female	87	15.23 (11.68, 18.84)	8.03 (5.84–8.68)	35.60 (27.52–44.61)
	≥50 years	Male	94	17.25 (13.76, 21.35)	9.23 (7.53–10.26)	46.35 (31.34–66.11)
		Female	83	18.45 (14.14, 22.87)	9.34 (7.32–11.25)	52.90 (34.72–54.43)
PLP/PL	< 50 years	Male	103	2.57 (1.99, 3.17)	1.16 (0.93–1.48)	4.23 (3.88–4.66)
		Female	87	2.55 (2.18, 2.88)	1.47 (0.97–1.77)	4.06 (3.42–4.18)
	≥50 years	Male	94	2.28 (1.90, 2.68)	1.27 (1.17–1.39)	3.34 (3.18–4.09)
		Female	83	2.41 (1.96, 2.78)	1.39 (1.35–1.59)	3.56 (3.37–3.60)
PLP/PA	< 50 years	Male	103	2.42 (1.68, 3.12)	0.76 (0.59–1.14)	5.15 (4.35–5.45)
		Female	87	2.60 (1.74, 3.33)	1.27 (0.88–1.41)	5.73 (4.51–7.62)
	≥50 years	Male	94	1.96 (1.38, 2.49)	0.57 (0.43–1.00)	3.99 (3.46–6.12)
		Female	83	2.41 (1.72, 3.13)	1.08 (0.95–1.37)	4.76 (3.79–5.73)
PAr	< 50 years	Male	103	0.32 (0.24, 0.40)	0.15 (0.15–0.18)	0.69 (0.58–0.86)
		Female	87	0.30 (0.22, 0.37)	0.14 (0.10–0.17)	0.54 (0.48–0.56)
	≥50 years	Male	94	0.38 (0.28, 0.47)	0.19 (0.13–0.21)	1.03 (0.66–1.37)
		Female	83	0.32 (0.24, 0.39)	0.16 (0.13–0.19)	0.57 (0.47–0.61)

The robust method based on Horn's algorithm was used to calculate 95% reference intervals (P2.5–P97.5), and their stability was validated via nonparametric bootstrap resampling.

PLP, pyridoxal 5′-phosphate; PL, pyridoxal; PA, 4-pyridoxic acid.

File Supplementary Figure 1 was erroneously published with the original version of this paper. The file has now been replaced.

The original version of this article has been updated.

