# Supplementary figures and images for: Age- and sex-specific reference intervals and determinants of plasma vitamin B6 metabolites in healthy Chinese adults
Source: Front Nutr. 2026 Feb 17;13:1782217. doi: 10.3389/fnut.2026.1782217 (PMC12954621; doi:10.3389/fnut.2026.1782217)

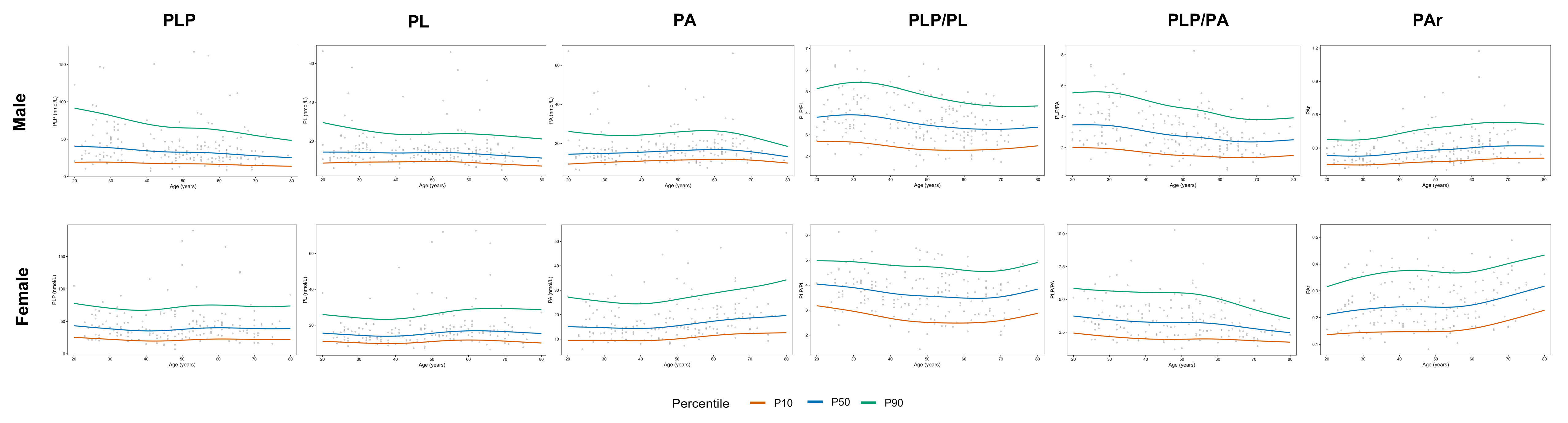

Supplement: Supplementary file 1 [file Image_1.TIF]
