# Supplementary material for: Age- and sex-specific reference intervals and determinants of plasma vitamin B6 metabolites in healthy Chinese adults
Source: Front Nutr. 2026 Feb 17;13:1782217. doi: 10.3389/fnut.2026.1782217 (PMC12954621; doi:10.3389/fnut.2026.1782217)
Supplement: Supplementary file 2 [file Table_1.DOCX]

**Supplementary Table 1**. Multivariable linear regression in males.

| **Variable** | **PLP** | | **PL** | | **PA** | | **PLP/PL** | | **PLP/PA** | | **PAr** | | |
| --- | --- | --- | --- | --- | --- | --- | --- | --- | --- | --- | --- | --- | --- |
|  | **β (95%CI)** | ***P* value** | **β (95%CI)** | ***P* value** | **β (95%CI)** | ***P* value** | **β (95%CI)** | ***P* value** | **β (95%CI)** | ***P* value** | **β (95%CI)** | ***P* value** |  |
| **Age (years)** | 0.007 (-0.003–0.017) | 0.154 | 0.006 (-0.002–0.014) | 0.145 | 0.001 (-0.006–0.009) | 0.736 | 0.001 (-0.003–0.006) | 0.580 | 0.006 (-0.002–0.014) | 0.132 | -0.006 (-0.012–0.001) | 0.114 |  |
| **BMI (kg/m²)** | 0.026 (-0.016–0.068) | 0.223 | 0.009 (-0.025–0.042) | 0.614 | 0.019 (-0.012–0.050) | 0.237 | 0.017 (-0.002–0.037) | 0.086 | 0.007 (-0.025–0.040) | 0.661 | -0.003 (-0.032–0.026) | 0.843 |  |
| **ALP (U/L)** | -0.007 (-0.013–-0.001) | **0.024** | -0.001 (-0.006–0.004) | 0.752 | -0.001 (-0.006–0.004) | 0.698 | -0.006 (-0.009–-0.003) | **<0.001** | -0.006 (-0.011–-0.001) | **0.012** | 0.005 (0.001–0.009) | **0.028** |  |
| **ALT (U/L)** | 0.001 (-0.007–0.010) | 0.797 | 0.003 (-0.003–0.010) | 0.332 | 0.000 (-0.006–0.006) | 0.967 | -0.002 (-0.006–0.002) | 0.273 | 0.001 (-0.006–0.008) | 0.772 | -0.001 (-0.007–0.004) | 0.622 |  |
| **eGFR (mL/min/1.73²)** | 0.007 (-0.002–0.017) | 0.129 | 0.007 (-0.000–0.015) | 0.063 | -0.003 (-0.010–0.005) | 0.477 | 0.000 (-0.004–0.005) | 0.948 | 0.010 (0.002–0.017) | **0.010** | -0.010 (-0.017–-0.003) | **0.004** |  |
| **CRP (mg/L)** | -0.025 (-0.162–0.112) | 0.719 | 0.003 (-0.106–0.112) | 0.958 | 0.041 (-0.060–0.143) | 0.420 | -0.028 (-0.093–0.037) | 0.396 | -0.066 (-0.173–0.040) | 0.220 | 0.063 (-0.033–0.159) | 0.194 |  |
| **Calcium (mmol/L)** | 0.914 (-0.569–2.397) | 0.224 | 1.554 (0.368–2.739) | **0.011** | 0.265 (-0.836–1.366) | 0.634 | -0.640 (-1.341–0.062) | 0.073 | 0.649 (-0.508–1.806) | 0.268 | -0.809 (-1.846–0.228) | 0.125 |  |
| **Phosphorus (mmol/L)** | 0.670 (-0.177–1.517) | 0.120 | -0.155 (-0.832–0.522) | 0.651 | 0.022 (-0.607–0.651) | 0.945 | 0.825 (0.424–1.226) | **<0.001** | 0.648 (-0.013–1.309) | 0.055 | -0.439 (-1.032–0.153) | 0.145 |  |
| **Albumin (g/L)** | 0.045 (-0.005–0.096) | 0.077 | 0.000 (-0.040–0.040) | 0.996 | 0.014 (-0.023–0.052) | 0.452 | 0.045 (0.021–0.069) | **<0.001** | 0.031 (-0.008–0.070) | 0.119 | -0.021 (-0.056–0.015) | 0.249 |  |
| Note: Outcomes were log-transformed. Reported β are unstandardized coefficients (original predictor units). Statistical significance was defined as two-tailed *P*<0.05, with significant results bold. PAr = PA/(PLP+PL). Abbreviations: BMI, body mass index; ALP, alkaline phosphatase; ALT, alanine aminotransferase; CRP: C-reactive protein; PLP, pyridoxal 5′-phosphate; PL, pyridoxal; PA, 4-pyridoxic acid. | | | | | | | | | | | | | |

**Supplementary Table 2**. Multivariable linear regression in females.

| **Variable** | **PLP** | | **PL** | | **PA** | | **PLP/PL** | | **PLP/PA** | | **PAr** | | |
| --- | --- | --- | --- | --- | --- | --- | --- | --- | --- | --- | --- | --- | --- |
|  | **β (95%CI)** | ***P* value** | **β (95%CI)** | ***P* value** | **β (95%CI)** | ***P* value** | **β (95%CI)** | ***P* value** | **β (95%CI)** | ***P* value** | **β (95%CI)** | ***P* value** |  |
| **Age (years)** | 0.008 (-0.003–0.019) | 0.162 | 0.004 (-0.006–0.014) | 0.411 | 0.003 (-0.007–0.012) | 0.590 | 0.004 (-0.000–0.008) | 0.064 | 0.005 (-0.002–0.013) | 0.161 | -0.005 (-0.012–0.003) | 0.210 |  |
| **BMI (kg/m²)** | -0.016 (-0.055–0.024) | 0.439 | -0.019 (-0.053–0.015) | 0.267 | -0.006 (-0.040–0.028) | 0.721 | 0.004 (-0.011–0.019) | 0.633 | -0.010 (-0.036–0.017) | 0.483 | 0.011 (-0.015–0.036) | 0.406 |  |
| **ALP (U/L)** | -0.004 (-0.011–0.003) | 0.237 | 0.004 (-0.002–0.009) | 0.227 | 0.002 (-0.004–0.008) | 0.514 | -0.008 (-0.010–-0.005) | **<0.001** | -0.006 (-0.010–-0.001) | **0.012** | 0.004 (-0.000–0.009) | 0.056 |  |
| **ALT (U/L)** | -0.002 (-0.011–0.007) | 0.636 | -0.004 (-0.012–0.004) | 0.278 | -0.005 (-0.012–0.003) | 0.247 | 0.002 (-0.001–0.006) | 0.223 | 0.002 (-0.004–0.008) | 0.451 | -0.002 (-0.008–0.004) | 0.525 |  |
| **eGFR (mL/min/1.73²)** | 0.004 (-0.007–0.014) | 0.501 | 0.001 (-0.008–0.010) | 0.790 | -0.008 (-0.017–0.001) | 0.076 | 0.002 (-0.002–0.006) | 0.236 | 0.012 (0.005–0.019) | **0.002** | -0.011 (-0.018–-0.004) | **0.001** |  |
| **CRP (mg/L)** | -0.029 (-0.190–0.133) | 0.725 | -0.042 (-0.181–0.096) | 0.546 | -0.070 (-0.207–0.066) | 0.309 | 0.014 (-0.047–0.074) | 0.657 | 0.042 (-0.067–0.151) | 0.449 | -0.040 (-0.143–0.064) | 0.447 |  |
| **Calcium (mmol/L)** | 0.745 (-0.627–2.117) | 0.283 | 0.738 (-0.438–1.913) | 0.215 | 0.125 (-1.036–1.287) | 0.831 | 0.007 (-0.507–0.522) | 0.977 | 0.620 (-0.306–1.546) | 0.187 | -0.619 (-1.497–0.260) | 0.165 |  |
| **Phosphorus (mmol/L)** | 0.706 (-0.257–1.669) | 0.149 | -0.078 (-0.903–0.747) | 0.851 | 0.078 (-0.737–0.894) | 0.849 | 0.784 (0.423–1.145) | **<0.001** | 0.628 (-0.023–1.278) | 0.058 | -0.455 (-1.072–0.161) | 0.146 |  |
| **Albumin (g/L)** | 0.027 (-0.028–0.082) | 0.326 | 0.001 (-0.046–0.048) | 0.957 | -0.018 (-0.065–0.028) | 0.437 | 0.026 (0.005–0.046) | **0.014** | 0.045 (0.008–0.082) | **0.017** | -0.039 (-0.075–-0.004) | **0.028** |  |
| Note: Outcomes were log-transformed. Reported β are unstandardized coefficients (original predictor units). Statistical significance was defined as two-tailed *P*<0.05, with significant results bold. PAr = PA/(PLP+PL). Abbreviations: BMI, body mass index; ALP, alkaline phosphatase; ALT, alanine aminotransferase; CRP: C-reactive protein; PLP, pyridoxal 5′-phosphate; PL, pyridoxal; PA, 4-pyridoxic acid. | | | | | | | | | | | | | |

**Supplementary Table 3.** Sex-stratified comparisons of independent determinants of vitamin B6 biomarkers between participants aged <50 and ≥50 years.

| **Variable** |  | **Males** | |  | |  | | **Females** |  | | |
| --- | --- | --- | --- | --- | --- | --- | --- | --- | --- | --- | --- |
|  | **<50 years** | | **≥50years** | | ***P* value** | | **<50 years** | **≥50years** | | ***P* value** |  |
| **ALP (U/L)** | 71.00 (62.00–79.00) | | 72.50 (63.75–84.25) | | 0.207 | | 58.00 (51.00–66.00) | 82.00 (67.00–90.00) | | **<0.001** |  |
| **eGFR (mL/min/1.73²)** | 99.15 (88.44–109.78) | | 87.41 (79.28–93.87) | | **<0.001** | | 106.82 (98.13–113.01) | 88.40 (79.12–97.33) | | **<0.001** |  |
| **Phosphorus (mmol/L)** | 1.07 ± 0.16 | | 0.99 ± 0.10 | | **0.005** | | 1.14 ± 0.14 | 1.15 ± 0.09 | | 0.694 |  |
| **Albumin (g/L)** | 47.23 ± 2.45 | | 45.24 ± 2.02 | | **<0.001** | | 45.59 ± 2.39 | 45.07 ± 2.24 | | 0.147 |  |

Note: Statistical significance was defined as two-tailed *P*<0.05, with significant results bold. Abbreviations: ALP, alkaline phosphatase.
